# A CPU–NPU Heterogeneous Edge Fault Diagnosis Framework for Industrial Sensor Data

**DOI:** 10.3390/s26165125

**Published:** 2026-08-13

**Authors:** Kangli Xu, Haozhou Wang, Chao Li, Hongxuan Liu, Chunxiao Xing

**Affiliations:** 1 Department of Computer Science and Technology, Tsinghua University, Beijing 100084, China; xukl21@mails.tsinghua.edu.cn (K.X.); xingcx@tsinghua.edu.cn (C.X.); 2 Beijing National Research Center for Information Science and Technology, Tsinghua University, Beijing 100084, China; wanghaozhou@mail.tsinghua.edu.cn (H.W.); hongxuan-liu@mail.tsinghua.edu.cn (H.L.)

**Keywords:** fault diagnosis, CPU–NPU heterogeneous computing, industrial sensors, edge devices, local inference, deep learning

## Abstract

Industrial sensor-based fault diagnosis often requires continuous data acquisition, local data processing, and timely model inference on edge devices. Although deep learning-based diagnostic methods have achieved promising performance, many existing approaches rely on cloud-centered processing pipelines that introduce communication overhead and potential data privacy concerns. This paper presents a CPU–NPU heterogeneous edge fault diagnosis framework for industrial sensor data. The framework runs on an RK3588 local edge device and includes SQLite- and RingBuffer-based data management, sliding window generation, micro-batch construction, and model inference. The CPU is responsible for data access and buffering, preprocessing, and micro-batch preparation, while the NPU executes fault diagnosis models using the RKNN runtime environment. By performing inference locally, the framework reduces the continuous transmission of raw sensor data and supports real-time fault diagnosis under resource-constrained edge devices. Experimental results demonstrate high consistency between ONNX-based CPU inference and RKNN-based NPU inference after model conversion. Furthermore, the effects of different data input paths and micro-batch configurations are systematically evaluated. A cross-platform comparison between server-class CPU/GPU execution and embedded NPU deployment is also conducted in terms of latency, throughput, and energy efficiency. The results show that RingBuffer-based streaming input significantly reduces data access overhead, while the effectiveness of NPU acceleration depends on both model structure and micro-batch size. The cross-platform results further demonstrate the energy efficiency advantages of the RK3588 platform, making it more suitable for practical deployment in resource-constrained edge scenarios. These findings provide practical insights for deploying fault diagnosis models on heterogeneous edge devices.

## 1. Introduction

Industrial equipment is monitored through high-frequency sensor data, making timely fault diagnosis important for equipment safety and maintenance. However, industrial equipment may experience wear and performance degradation, leading to equipment failures and safety incidents. Therefore, industrial maintenance requires the early identification of potential risks and the rapid detection of faults once they occur [1,2]. Traditional methods relying on expert experience are difficult to address complex conditions and real-time monitoring. The operation of industrial equipment is complex, and the data are mainly time-series sensor data with high sampling frequencies and large volumes, bringing challenges for intelligent fault diagnosis.

Bearings are critical components in industrial equipment, and their operating conditions directly affect system reliability and safety [3]. Machine learning and deep learning methods have therefore been widely applied for industrial equipment health monitoring. Early studies mainly relied on handcrafted time-domain and frequency-domain features combined with traditional machine learning methods such as support vector machines (SVM) [4] and K-means clustering [5]. Although these methods can perform well in specific scenarios, their effectiveness usually depends on handcrafted features and domain expertise.

More recently, deep learning models such as Convolutional Neural Networks (CNNs) [6] and Recurrent Neural Networks (RNNs) [7] have been introduced for bearing fault diagnosis, enabling automatic feature extraction from sensor signals and improved diagnostic performance. Transformer-based architectures have also been explored for time-series analysis because self-attention can model long-range temporal dependencies [8]. However, the computational and memory requirements of Transformer models often limit their deployment on resource-constrained edge devices. Recent studies have shown that lightweight models such as CNNs and RNNs can achieve competitive fault diagnosis performance with lower computational requirements, making them suitable candidates for edge deployment scenarios [9].

Deploying intelligent fault diagnosis models directly on edge devices remains challenging due to their limited computational and storage resources [10]. Therefore, cloud-based centralized processing is commonly used. However, cloud-based methods still have limitations. The first challenge is the additional latency introduced by data transmission. Uploading large volumes of high-frequency sensor data to the cloud can introduce transmission latency, which may delay fault detection. In some industrial scenarios, unstable network conditions or limited bandwidth may lead to increased latency, packet loss and retransmission problems [11,12]. Second, cloud-based processing may expose industrial data to privacy and security risks during transmission and storage. Meanwhile, encrypting the data at the edge side also provides additional computational overhead, increasing the overall system burden.

The rapid development of the Industrial Internet of Things (IIoT) and edge computing have promoted the deployment of computing resources closer to industrial equipment. By performing data processing and model inference locally, edge computing can reduce communication overhead, improve response latency, and alleviate privacy concerns associated with cloud-centric architectures [13].

Although large parameter models are difficult to deploy on embedded edge devices [14], lightweight fault diagnosis models provide a feasible option for local inference. Meanwhile, modern edge devices now integrate Neural Processing Units (NPUs), which provide dedicated hardware acceleration for deep learning inference and further improve the feasibility of edge-side deployment. However, deploying fault diagnosis models on edge devices still requires design between data access, preprocessing, batching, and hardware-accelerated inference. Moreover, deploying deep learning models on NPUs requires model conversion, runtime adaptation, and consistency evaluation after conversion.

To address these issues, this paper presents a CPU–NPU heterogeneous edge fault diagnosis framework for industrial sensor data. The main contributions of this work are summarized as follows:

1. Design an embedded CPU–NPU heterogeneous deployment framework for resource-constrained edge fault diagnosis, in which the CPU manages sensor data access, sliding window generation, and micro-batch preparation, while the NPU performs local model inference, enabling effective utilization of the heterogeneous CPU–NPU architecture.
2. Deploy CNN, RNN and TCN fault diagnosis models on an edge NPU device through an ONNX-to-RKNN conversion workflow and evaluate the consistency between ONNX-based CPU inference and RKNN-based NPU inference.
3. Evaluate how SQLite- and RingBuffer-based data access paths and different micro-batch sizes affect data preparation overhead, inference latency, throughput, and end-to-end pipeline latency.

## 2. Related Work

### 2.1. AI-Based Bearing Fault Diagnosis

Early bearing fault diagnosis approaches combined handcrafted features with machine learning algorithms, including K-means, PCA, and KNN [15,16,17]. Although these methods are interpretable, their performance often depends on handcrafted features and domain expertise, which may limit their use in complex operating conditions.

Deep learning models such as Convolutional Neural Networks (CNNs) and Recurrent Neural Networks (RNNs) have been widely used for fault diagnosis as they can learn more complex fault-related patterns from raw vibration signals [18,19]. This reduces the dependence on manually designed time-domain and frequency-domain features.

Recently, Transformer-based architectures have also been introduced for time-series fault diagnosis tasks because of their capability to model long-range temporal dependencies through self-attention mechanisms [8,20]. Although these models have demonstrated promising performance, their computational and memory requirements can limit deployment on resource-constrained edge devices. For edge deployment, lightweight CNN and RNN models remain useful because they can provide competitive diagnosis performance with lower computational cost [9].

### 2.2. Edge-Based Fault Diagnosis and Deployment

In IIoT-based fault diagnosis systems, cloud platforms are often used for data storage, model training, and fault analysis [21], while edge computing enables data processing and inference closer to the data source.

Recent studies have explored edge-side fault diagnosis for industrial equipment by deploying lightweight models on edge devices. For example, some CNN-based models have been applied to industrial fault diagnosis on edge devices, showing the potential of local inference for real-time monitoring [22,23,24]. Other works improve deployability through lightweight network design, model compression, knowledge distillation, or quantization [25,26,27].

Deploying fault diagnosis models on edge devices involves not only model design but also the execution hardware and software framework. Recent studies have shown that CPU, GPU, and NPU platforms can produce different latency and throughput results under different model workloads [28]. In addition, model deployment on edge accelerators typically relies on hardware-specific execution frameworks and model conversion toolchains, which can further affect inference performance and deployment complexity. Therefore, it is necessary to evaluate the deployment behavior of fault diagnosis models under different edge execution settings.

### 2.3. NPU-Accelerated Edge Inference

Embedded edge devices now increasingly integrate AI accelerators for local inference. Unlike CPUs, Neural Processing Units (NPUs) provide dedicated hardware support for deep learning inference, and they are increasingly used in embedded edge devices [28].

Several studies have deployed deep learning models on edge devices equipped with NPUs. For example, AI models have been deployed on RK3588 platforms for industrial fault detection using NPU acceleration [29].

## 3. CPU–NPU Heterogeneous Edge Fault Diagnosis Framework

### 3.1. System Overview

Industrial fault diagnosis systems often involve many edge devices deployed close to equipment. Deploying edge devices with high-performance computing hardware can increase deployment cost and bring high maintenance expenses, which is difficult to promote in real industrial scenarios. Thus, edge-side fault diagnosis usually needs to operate under limited computing resources. Therefore, this work develops a CPU–NPU heterogeneous edge fault diagnosis framework for resource-constrained embedded edge devices.

Fault diagnosis based on industrial sensor signals can be formulated as a time-series classification task. In many practical scenarios, lightweight models are suitable candidates because they reduce computational overhead while retaining diagnostic capability.

In this work, diagnostic models are trained offline and then deployed on edge devices for inference. During deployment, the edge device only performs local data processing and model inference.

**Figure 1:**
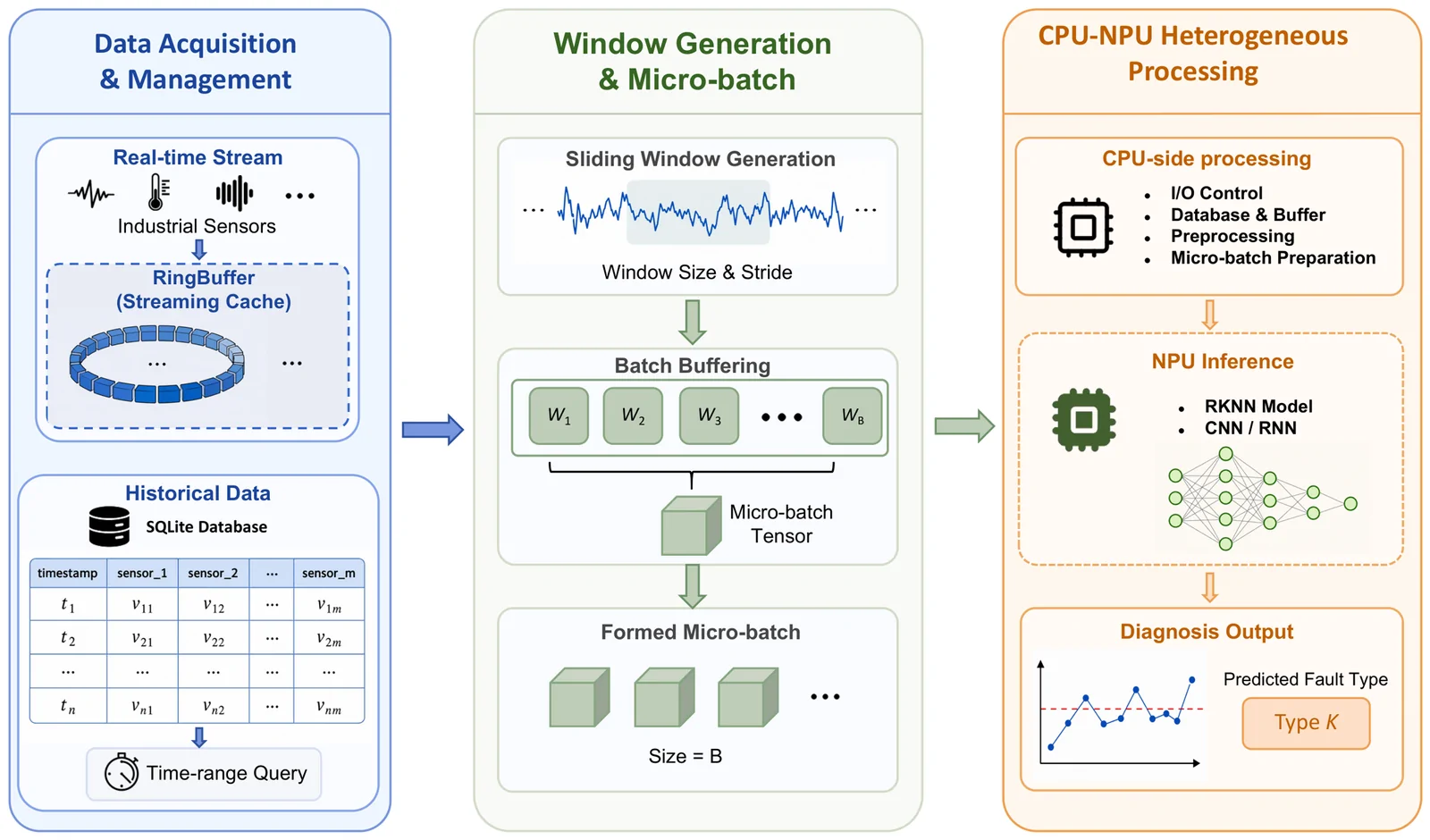
Overall framework of the CPU–NPU heterogeneous edge fault diagnosis pipeline.

The proposed framework focuses on combining CPU-side data management and preprocessing with NPU-side inference for resource-constrained embedded edge deployment. The framework is designed for deployment on resource-constrained embedded edge devices with heterogeneous computing architectures. In this work, RK3588 is used as the target deployment platform for evaluation. The framework focuses on edge-side deployment behavior, including data access overhead, micro-batch preparation, NPU inference latency, and end-to-end pipeline latency. As shown in Figure 1, the overall workflow consists of three stages: data acquisition and management, window generation and micro-batch construction, and CPU–NPU heterogeneous inference and output generation.

At the data level, the framework supports both real-time sensor data streams and historical sensor data. Real-time data are buffered using a RingBuffer to emulate online diagnosis under continuous data streams. Historical data are stored in a local SQLite database and accessed through range queries for offline diagnosis. Both input paths provide one-dimensional time-series signals as the input to the sliding window generation module.

At the computation level, this work uses a CPU and NPU collaborative approach. The CPU is used for data access, buffer management, sliding window generation, input formatting, and micro-batch preparation. The NPU is responsible for executing the converted RKNN model and produces fault diagnosis results.

**Figure 2:**
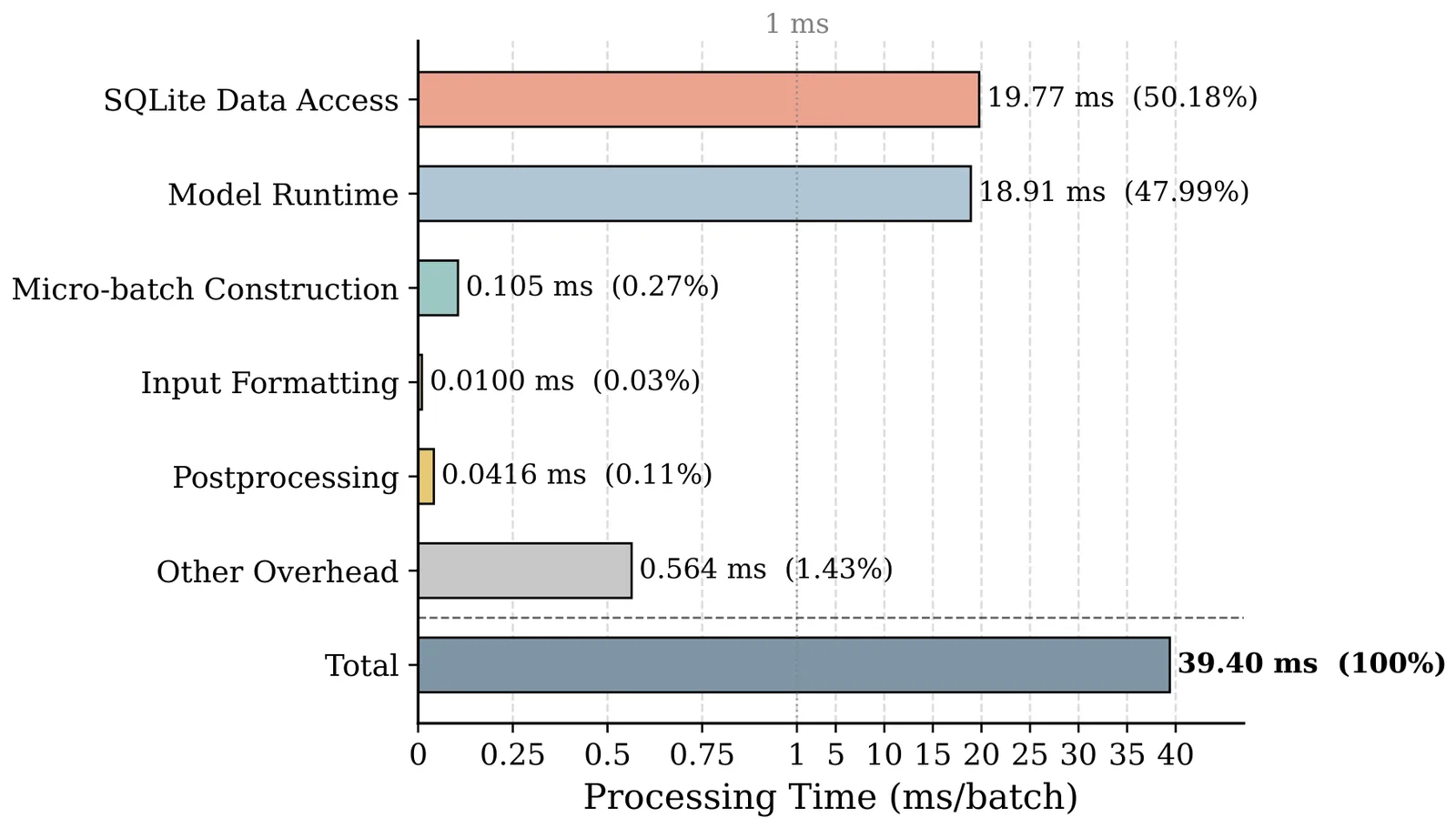
Latency breakdown of the CPU-only CNN fault diagnosis pipeline with SQLite data access on the evaluated edge device. The horizontal axis uses piecewise scaling to improve the visibility of small latency components.

To verify the rationality of the heterogeneous task partitioning, we conduct a latency breakdown test on the CPU-only fault diagnosis framework running on edge devices. The measurement adopts a CNN model and reads data via SQLite. As illustrated in Figure 2, SQLite data access and model execution account for 50.18% and 47.99% of the total processing time. These results show that model execution still consumes considerable CPU resources even for lightweight models. Therefore, offloading model inference to the NPU can free up more CPU resources for database access, buffering, window generation and input preparation.

At the deployment level, this work follows an offline training and online deployment strategy. The fault diagnosis models are first trained on a computing platform with sufficient resources and then exported to the ONNX format. The ONNX models are then converted into RKNN models to run on the NPU through the RKNN Runtime. During fault detection, the CPU prepares model inputs and manages the execution workflow, and the NPU performs model inference through the RKNN Runtime. This design separates model training from edge-side inference and allows the edge device to focus on local data processing and fault diagnosis.

### 3.2. Data Acquisition and Management

Industrial sensor signals are continuous time-series data and usually have a high sampling frequency. To support both online diagnosis for streaming data and offline diagnosis for historical data, this work uses two data input paths: a SQLite-based historical input path and a RingBuffer-based streaming input path.

In both input paths, continuous sensor signals are divided into fixed-length windows before model inference. The window length is predefined during model training and preprocessing. During deployment, the window length remains fixed so that the two input paths can generate inputs with the same shape.

For the SQLite-based historical data path, sensor signals are first stored in a local SQLite database. In the experiments, two SQLite access methods are considered. The first is per-window SELECT, in which a database query is executed for each sliding window. This method represents direct window-by-window access from the database. The second is segment-level SELECT followed by in-memory slicing, in which a continuous signal segment is first queried from the database and then divided into sliding windows in memory. This method is used to analyze how reducing the number of database queries affects window generation overhead.

For the real-time data path, a RingBuffer is used as a streaming cache structure. The capacity of the RingBuffer is set to the window length, which means that the buffer stores exactly the number of samples required for one complete input window. When a new sample arrives, it is written to the next position of the circular buffer. If the buffer is already full, the oldest sample is overwritten by the new sample. This circular overwrite mechanism ensures that the RingBuffer always keeps the most recent sensor samples.

Once the RingBuffer is full and the current sample position satisfies the predefined stride condition, a complete window is extracted from the buffer. Since the RingBuffer only maintains a fixed number of recent samples, its memory usage is fixed, and it avoids frequent database queries during online window generation. Therefore, the RingBuffer-based input path is suitable for low-overhead window generation in streaming sensor data scenarios.

### 3.3. Window Generation and Micro-Batch Construction

The fault diagnosis task is treated as a time-series classification task. Given a sensor signal sequence $X=\{x_{1},x_{2},\ldots ,x_{T}\}$ of length *T* and *K* fault categories, the edge model predicts a fault label Y∈{1,2,…,K} from the input signal.

To enable fixed-length model inputs, continuous sensor signals are segmented using a sliding window strategy. Each input window is defined as

(1) $$X_{t}={\left[x_{t},x_{t+1},\ldots ,x_{t+L-1}\right]}^{⊤}\in R^{L\times C},$$

where $X_{t}$ denotes a signal segment starting at time index *t*, *L* denotes the window length, and *C* denotes the number of the signal channels. For a signal sequence of length *T*, the number of valid windows can be calculated as

(2) $$N_{w}=\left\lfloor \frac{T-L}{S}\right\rfloor +1,$$

where *S* denotes the stride. This equation shows that the stride directly affects the number of generated windows and therefore influences the workload of the edge inference framework.

For the real-time input path, Algorithm 1 describes how streaming sensor data is accumulated in the RingBuffer, divided into sliding windows, and packed into micro-batches for inference. The RingBuffer has a fixed capacity equal to the window length *L* (Line 1). When a new sample arrives, it is appended to the RingBuffer (Line 5). If the buffer is already full, the oldest sample is overwritten by the newest one. A sliding window is extracted only when the RingBuffer contains enough samples and the stride condition is satisfied (Lines 7–8). Therefore, the RingBuffer maintains the most recent *L* samples and has fixed memory usage.
**Algorithm 1** RingBuffer-based micro-batch construction and inference**Require:** Sensor stream $X=\{x_{1},x_{2},\ldots ,x_{T}\}$, window length *L*, stride *S*, micro-batch size *B*, model input layout *R*, inference runtime F**Ensure:** Predicted fault labels $\hat{Y}$  1:Initialize RingBuffer R with capacity *L*  2:Initialize batch buffer B←Ø  3:Initialize prediction list $\hat{Y}\leftarrow Ø$  4:**for** each incoming sample $x_{t}$ in X **do**  5:      Append $x_{t}$ to R  6:      **if** R is full and (t−L)modS=0 **then**  7:            Extract a sliding window $W_{i}$ from R  8:            Append $W_{i}$ to the batch buffer B  9:      **end if**10:      **if** |B|=B **then**11:            Construct a micro-batch tensor *M* from B12:            Reshape *M* according to the model input layout *R*13:            Convert *M* to a contiguous float32 array14:            Obtain prediction labels ${\hat{Y}}_{B}=F\left(M\right)$15:            Append ${\hat{Y}}_{B}$ to $\hat{Y}$16:            Clear the batch buffer B17:      **end if**18:**end for**19:**return** $\hat{Y}$

The generated windows are temporarily stored in a batch buffer B. Once the number of buffered windows reaches the predefined batch size *B*, the CPU constructs them into a micro-batch tensor and performs the input preparation (Lines 11–13). The prepared micro-batch is then submitted to the NPU model for fault diagnosis (Line 14). After inference, the batch buffer is cleared for the next cycle. In this process, the CPU maintains the input stream and constructs micro-batches, while the NPU executes the model inference.

The micro-batch size affects the online response latency. To construct a micro-batch containing *B* sliding windows, the number of sensor samples required by the streaming input path ($N_{\mathrm{sample}}$) and the maximum time required to accumulate micro-batches containing *B* windows ($T_{\mathrm{batch}}$) are defined as

(3) $$N_{\mathrm{sample}}=L+\left(B-1\right)S,$$

(4) $$T_{\mathrm{batch}}=\frac{N_{\mathrm{sample}}}{f_{s}}=\frac{L+(B-1)S}{f_{s}},$$

where *B* denotes the micro-batch size, *L* denotes the window length, *S* denotes the stride and $f_{s}$ denotes the sensor sampling frequency.

For example, for the CWRU sampling frequency of 12 kHz, L=128, S=64, batch sizes of 128 and 2048 require approximately 0.688 s and 10.928 s of accumulation time. A batch size of 128 provides low latency under this setting, which can provide second-level response latency for online diagnostic scenarios. In contrast, a larger micro-batch size increases the accumulation delay and extends the diagnosis response time and should therefore be selected according to the sampling frequency and response-time requirements of the target application.

### 3.4. CPU–NPU Inference Pipeline

**Figure 3:**
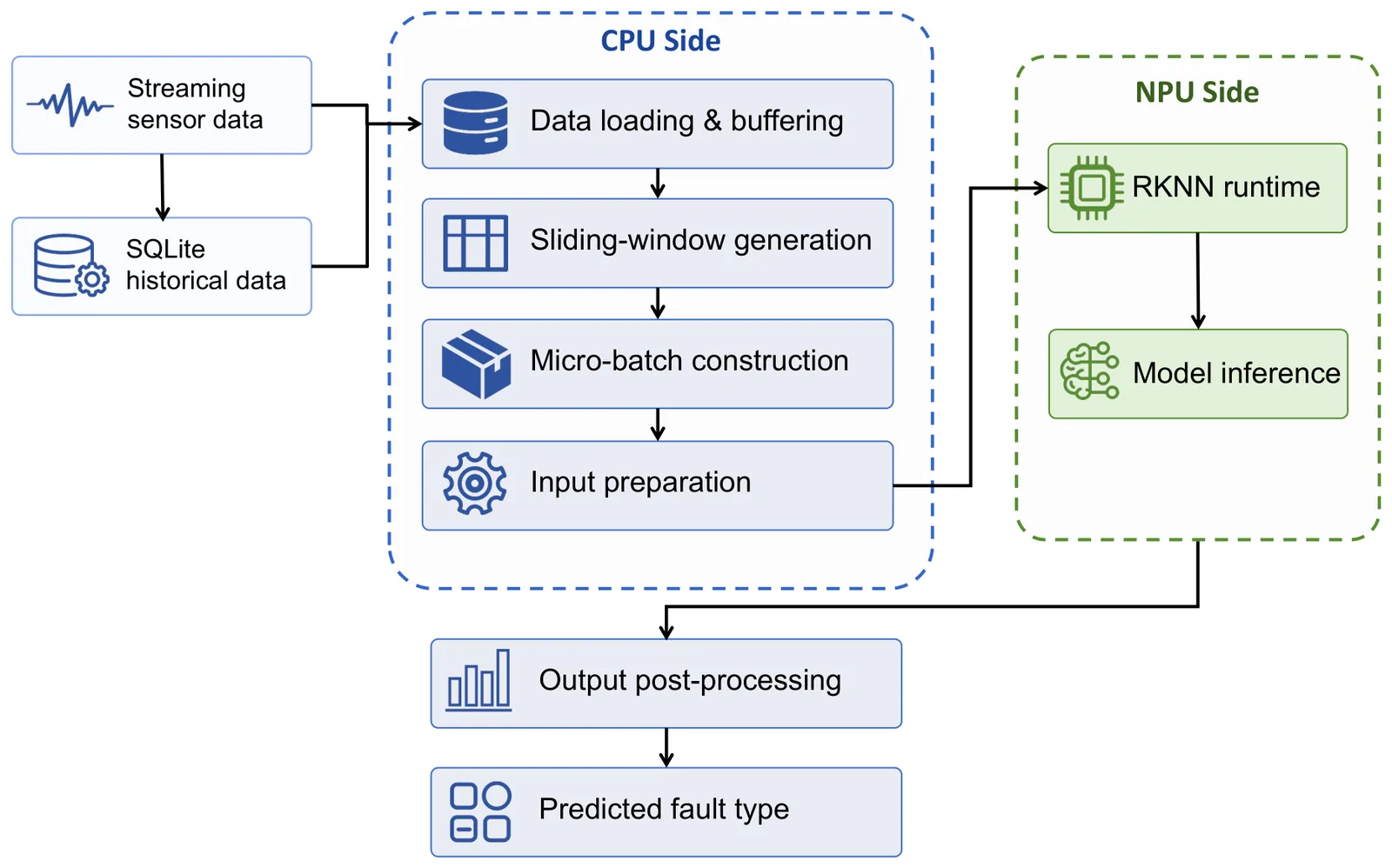
Runtime workflow of the CPU–NPU heterogeneous inference pipeline.

The deployment pipeline is designed for lightweight time-series models used in edge-side fault diagnosis. Figure 3 shows the runtime workflow of the CPU–NPU heterogeneous inference pipeline.

In the proposed heterogeneous inference pipeline, the CPU and NPU perform different tasks. The CPU is responsible for data-side and input-side processing, including data loading, SQLite queries, RingBuffer management, sliding window generation, micro-batch construction, input preparation and post-processing.

The runtime workflow starts from local data loading. For historical diagnosis, the CPU reads the sensor data from the SQLite database. For online diagnosis, incoming sensor samples are continuously written into the RingBuffer. When new samples arrive, the RingBuffer updates its stored data and maintains the most recent signal segment for window sliding.

After the data are loaded, the CPU converts the continuous sensor signal into fixed-length windows. In the SQLite-based path, sliding windows are generated from the queried data. In the RingBuffer-based path, windows are extracted from the RingBuffer. The generated windows are then appended to a batch buffer. This buffer stores multiple windows before inference so that the NPU can process them together.

When the batch buffer reaches the predefined micro-batch size, the CPU constructs a micro-batch tensor from the collected windows. The CPU then performs necessary input preparation before sending the data to the NPU, including optional normalization and tensor formatting. These steps ensure that the prepared input satisfies the execution requirements of the deployed RKNN model.

The trained models are first exported from PyTorch (v2.13.0) to the ONNX format and then converted to the RKNN format to support deployment on the NPU platform. During inference, the RKNN model is loaded through the RKNN Runtime, and the micro-batch tensor prepared by the CPU is sent to the model on NPU for inference. After inference, the output tensor is returned to the CPU for post-processing to obtain the predicted fault type.

To evaluate model conversion consistency and NPU inference behavior, this work implements both ONNX Runtime CPU inference and RKNN Runtime NPU inference. The CPU implementation serves as a baseline for deployment evaluation, while the RKNN implementation is used to analyze the deployment behavior and inference performance of fault diagnosis models on the embedded NPU platform.

## 4. Experimental Setup

### 4.1. Hardware Platforms

All edge-side deployment and inference experiments were conducted on an RK3588 edge device running Ubuntu 24.04. The platform is equipped with an 8-core CPU, 16 GB of memory, and an integrated NPU. The RK3588 platform is used as a low-cost embedded CPU–NPU device for local edge inference. The cost and power consumption of this device are relatively low, making it suitable for practical embedded deployment scenarios. For this device, ONNX Runtime (v1.23.2) [30] was used for CPU-based inference. RKNN Toolkit2 (v2.3.2) [31] was used to convert ONNX models into the RKNN format, while the RKNNLite API (v2.3.2) and RKNN Runtime (v2.3.2) were used to load and execute the converted models on the RK3588 NPU.

For local data management, SQLite (v3.45.1) was used to store historical sensor signals, and a RingBuffer structure was implemented for streaming sensor data. This configuration enables the evaluation of both historical data access and online inference scenarios on the edge device.

For cross-platform comparison, experiments were also conducted on a server equipped with two Intel Xeon Gold 6346 processors and an NVIDIA A100-SXM4-80 GB GPU. CPU inference on this server used the ONNX Runtime CPU Execution Provider, while GPU-accelerated inference used the CUDA Execution Provider.

### 4.2. Datasets

The experiments were conducted using two public bearing fault datasets: the Case Western Reserve University (CWRU) bearing fault dataset [32] and the Machinery Failure Prevention Technology (MFPT) bearing fault dataset [33]. In this work, the two datasets are used as the bearing-vibration fault diagnosis workloads for deployment evaluation.

The CWRU dataset contains vibration signals collected under normal conditions and multiple bearing fault conditions, including inner-race faults, outer-race faults, and rolling element faults with different fault severities. A 10-class classification task is adopted, and the processed dataset contains 7920 training samples and 1980 test samples.

The MFPT dataset contains vibration signals collected from normal and different fault conditions, including normal bearing conditions, outer-race faults and inner-race faults. In this work, these data are organized into a 3-class classification task. After preprocessing, the dataset contains 8400 training samples and 2100 test samples.

For both datasets, the vibration signals are normalized and then segmented into fixed-length samples using a sliding window strategy, and the window length is set to 128. The processed samples are divided into training and test sets at an 8:2 ratio. The generated windows are used as inputs for the evaluated fault diagnosis models.

### 4.3. Models and Deployment Configuration

This work uses three fault diagnosis models, including a CNN, an RNN and a temporal convolutional network (TCN) [34] to evaluate inference behavior on heterogeneous edge devices. The models are used as representative workloads for deployment evaluation. By comparing the deployment performance of CNN, RNN and TCN models, the influence of model structure on CPU and NPU inference behavior can be analyzed.

Table 1 summarizes the configurations of the three models used in the deployment experiments. The CNN model contains two one-dimensional convolutional layers, the RNN model contains one LSTM layer, and the TCN model consists of three residual blocks composed of dilated one-dimensional convolutions and residual connections. All models use an input length of 128. The output dimension is set to 10 for the CWRU dataset and 3 for the MFPT dataset.

All models were trained using the Adam optimizer and cross-entropy loss with a training batch size of 64. The random seed was set to 42, and the learning rate remained constant throughout each training run. The training configurations are summarized in Table 2.

The ONNX and RKNN size columns report the ONNX model size and the converted RKNN model size with a fixed batch size of 128. Since ONNX and RKNN use different model formats, the file size changes after model conversion. RKNN models are compiled and deployed for specific target platforms. During conversion, the computational graph is optimized and restructured for efficient execution on the RK3588 platform. Therefore, the size of an RKNN file depends not only on model parameters, but also on the compiled computational graph representation and input configurations.

All models are first trained offline using PyTorch and then exported to the ONNX format to support cross-platform deployment. For the CNN and TCN models, the FP32 ONNX models are converted into RKNN models and executed in FP16 precision on the RK3588 NPU. For the RNN consistency evaluation, the FP32 ONNX model is quantized into an INT8 ONNX model and an INT8 RKNN model. The INT8 ONNX model uses post-training static quantization with the QDQ format, MinMax calibration, and per-tensor signed INT8 weights and activations. The INT8 RKNN model is generated using the default INT8 quantization configuration of RKNN Toolkit2. For both CWRU and MFPT, the calibration data are constructed from the corresponding training split, using up to 1024 windows with a calibration batch size of 64. The generated RKNN models are deployed on the RK3588 NPU and executed through the RKNN Runtime.

For the RNN model, the standard ONNX LSTM operator is mapped to RKNN’s internal exLSTM operator. The runtime analysis shows that exLSTM is executed on the NPU with zero CPU execution time and accounts for approximately 95% of the total execution time, confirming that the LSTM computation does not fall back to the CPU.

For deployment evaluation, different batch sizes ranging from 1 to 2048 are evaluated to analyze the influence of micro-batch configuration on inference performance. For RKNN inference, these batch sizes are treated as predefined input configurations specified during model conversion.

### 4.4. Evaluation Methodology

To evaluate the proposed CPU–NPU heterogeneous edge fault diagnosis framework, four groups of experiments were conducted on the RK3588 platform. A cross-platform performance and energy efficiency experiment was also conducted to compare the RK3588 NPU with CPU and GPU execution on an NVIDIA A100 server.

Model conversion consistency is evaluated by comparing ONNX-based CPU inference and RKNN-based NPU inference. Accuracy, macro-F1 score, class consistency, and the number of inconsistent predictions are used to measure whether ONNX-to-RKNN conversion changes the predicted fault labels. The accuracy and macro-F1 score are defined as

(5) $$\mathrm{Accuracy}=\frac{N_{\mathrm{correct}\;\mathrm{predictions}}}{N_{\mathrm{samples}}},$$

(6) $$\begin{array}{c} {F1}_{\mathrm{macro}}=\frac{1}{K}\sum_{k=1}^{K}\frac{2\cdot {\mathrm{Precision}}_{k}\cdot {\mathrm{Recall}}_{k}}{{\mathrm{Precision}}_{k}+{\mathrm{Recall}}_{k}},\\ {\mathrm{Precision}}_{k}=\frac{{\mathrm{TP}}_{k}}{{\mathrm{TP}}_{k}+{\mathrm{FP}}_{k}},\\ {\mathrm{Recall}}_{k}=\frac{{\mathrm{TP}}_{k}}{{\mathrm{TP}}_{k}+{\mathrm{FN}}_{k}}, \end{array}$$

where $N_{\mathrm{correct}\;\mathrm{predictions}}$ denotes the number of correctly classified samples, $N_{\mathrm{samples}}$ denotes the total number of evaluated samples, and *K* denotes the number of fault classes. For class *k*, ${\mathrm{TP}}_{k}$, ${\mathrm{FP}}_{k}$, and ${\mathrm{FN}}_{k}$ denote the numbers of true positives, false positives, and false negatives. ${\mathrm{Precision}}_{k}$ and ${\mathrm{Recall}}_{k}$ represent the precision and recall for class *k*.

The class consistency between ONNX and RKNN inference results is defined as

(7) $$\mathrm{Consistency}=\frac{N_{\mathrm{same}}}{N_{\mathrm{samples}}}\times 100\%,$$

where $N_{\mathrm{same}}$ denotes the number of samples receiving identical predicted labels from ONNX and RKNN inference, and $N_{\mathrm{samples}}$ denotes the total number of evaluated samples.

Data input overhead is then evaluated using three input methods, including SQLite per-window SELECT, SQLite segment-level SELECT, and RingBuffer-based streaming input. On resource-limited edge devices, SQLite provides persistent local storage for historical sensor data, which cannot be fully stored in memory. The per-window SELECT method serves as a simple baseline to quantify the overhead of frequent database queries. The segment-level SELECT method uses a single range query to retrieve a continuous signal segment for window generation. The RingBuffer-based streaming input maintains in-memory buffering for real-time streaming data. Data access time, window generation time, and total processing overhead are measured and compared. The front-end overhead is defined as

(8) $$T_{\mathrm{front-end}}=T_{\mathrm{access}}+T_{\mathrm{window}},$$

where $T_{\mathrm{access}}$ denotes the average data access time per window and $T_{\mathrm{window}}$ denotes the average sliding window generation time per window. For the RingBuffer-based method, database access time is not included, so $T_{\mathrm{access}}$ is set to zero. For per-window SELECT, complete windows are directly retrieved from the database, so no additional in-memory window generation time is reported.

In this experiment, the window length was set to 128, and the stride was set to 64. A total of 1000 valid windows were evaluated for each input method. For SQLite segment-level SELECT, a continuous signal segment was first queried from the database. The segment length was calculated as

(9) $$L_{\mathrm{seg}}=L+\left(N_{w}-1\right)S,$$

where *L* denotes the window length, *S* denotes the stride, and $N_{w}$ denotes the number of evaluated windows. In this experiment, $L_{\mathrm{seg}}$ was 64,064 samples. This setting ensures that the queried segment can generate the same number of sliding windows as the other input methods.

To evaluate inference performance on the edge device, inference experiments are conducted under different micro-batch sizes from 128 to 2048, measuring latency, throughput, and speedup. To ensure statistical stability, the latency is averaged over 1000 iterations. Throughput and hardware utilization are measured during a 10 s continuous inference period. The large batch sizes are included to evaluate inference behavior as the micro-batch size increases. As described in Section 3.3, a batch size of 2048 may introduce additional latency in some online scenarios, so its practical application depends on the sampling frequency and response time requirements.

The latency, throughput, and speedup metrics are defined as

(10) $$\mathrm{Latency}=\frac{1}{N}\sum_{i=1}^{N}\left(t_{i}^{\mathrm{end}}-t_{i}^{\mathrm{start}}\right),$$

(11) $$\mathrm{Throughput}=\frac{N_{\mathrm{samples}}}{T_{\mathrm{inference}}},$$

(12) $$\mathrm{Speedup}=\frac{T_{\mathrm{CPU}}}{T_{\mathrm{NPU}}},$$

where *N* denotes the number of inference runs, $N_{\mathrm{samples}}$ denotes the number of processed samples, $T_{\mathrm{inference}}$ denotes the total inference time, $T_{\mathrm{CPU}}$ denotes the inference time of the ONNX model executed on the CPU, and $T_{\mathrm{NPU}}$ denotes the inference time of the RKNN model executed on the NPU.

End-to-end deployment performance is evaluated using three pipeline configurations, including CPU+SQLite, NPU+SQLite, and NPU+RingBuffer. The average latency per window and peak memory consumption are measured for each configuration.

The end-to-end latency includes all processing stages involved in a deployment pipeline, including data access or window generation, micro-batch construction, input preparation, data copy, and model inference. The average latency per window is defined as

(13) $$T_{e2e}=\frac{T_{\mathrm{pipeline}}}{N_{\mathrm{window}}},$$

where $T_{\mathrm{pipeline}}$ denotes the total execution time of the pipeline and $N_{\mathrm{window}}$ denotes the number of processed windows. Peak memory consumption is recorded during execution to evaluate the memory overhead introduced by different deployment configurations.

For this experiment, the selected CWRU signal was segmented using a window length of 128 and a stride of 64. The maximum number of windows was set to 4096 as an upper bound.

Finally, cross-platform inference performance and energy efficiency were evaluated using three execution configurations: CPU-only ONNX inference on the Intel Xeon server, GPU-accelerated ONNX inference on the NVIDIA A100 server, and RKNN-based NPU inference on the RK3588 device. The evaluated metrics include batch latency, per-sample latency, throughput, average measured power, throughput per watt, and energy consumption per sample. For the server experiments, each configuration was warmed up for 20 batches and then executed continuously for 60 s.

For the server platform, CPU power was calculated from energy measurements obtained through Intel RAPL, while GPU power was sampled using nvidia-smi.

The RK3588 device does not provide an interface for directly measuring board-level power. Therefore, the power consumption of the RK3588 platform was estimated using its maximum operating power. This estimated value was used for the energy-efficiency calculations of the RK3588 platform.

The evaluation metrics are defined as

(14) $$\mathrm{Throughput}\;\mathrm{per}\;\mathrm{watt}=\frac{\mathrm{Throughput}}{P_{T}},$$

(15) $$E_{s}=\frac{P_{T}}{\mathrm{Throughput}},$$

where $P_{T}$ denotes the power value used for the energy-efficiency calculation, and $E_{s}$ denotes the energy consumption per processed sample.

## 5. Results and Analysis

### 5.1. Model Conversion Consistency

The trained CNN, RNN, and TCN models were tested using ONNX Runtime on the CPU and RKNN Runtime on the RK3588 NPU to evaluate whether model conversion affects diagnostic performance. The evaluation was conducted on the CWRU and MFPT datasets. The results are reported in Table 3. This experiment focuses on whether the converted RKNN models maintain the original diagnostic accuracy and whether their predicted labels remain consistent with the ONNX models.

As shown in Table 3, the CNN and TCN models maintain highly stable diagnostic results after conversion. On the CWRU dataset, both models achieve identical accuracy and macro-F1 scores before and after conversion and no inconsistent predictions are found. Similar results are observed on the MFPT dataset. The TCN model maintains 100% class consistency, while the CNN model produces only one inconsistent prediction among 2100 test samples. The RNN model also shows high prediction consistency after conversion, although a small number of prediction differences can be observed. Specifically, the RKNN model produces 14 inconsistent predictions on the CWRU dataset and 12 inconsistent predictions on the MFPT dataset.

Since the CNN and RNN models produce several inconsistent predictions after conversion, a paired exact McNemar test was conducted to examine whether the prediction differences lead to statistically significant accuracy changes. The resulting *p*-value for CNN on MFPT is 1.00, and for RNN is 0.27 on CWRU and 1.00 on MFPT, indicating that no statistically significant accuracy difference is detected at the 0.05 significance level. The RNN prediction inconsistency may be related to reduced numerical precision and differences between the quantized INT8 ONNX and RKNN models. These numerical differences may accumulate across recurrent steps. In addition, implementation differences between ONNX LSTM and RKNN exLSTM may also lead to the observed differences.

Overall, the results show that the proposed ONNX-to-RKNN deployment workflow can execute the CNN, RNN and TCN fault diagnosis models on the RK3588 NPU while keeping high label consistency with ONNX-CPU inference. The converted models can be executed on the RK3588 NPU while maintaining acceptable diagnostic accuracy and high prediction consistency.

### 5.2. Data Access and Window Generation Overhead

**Figure 4:**
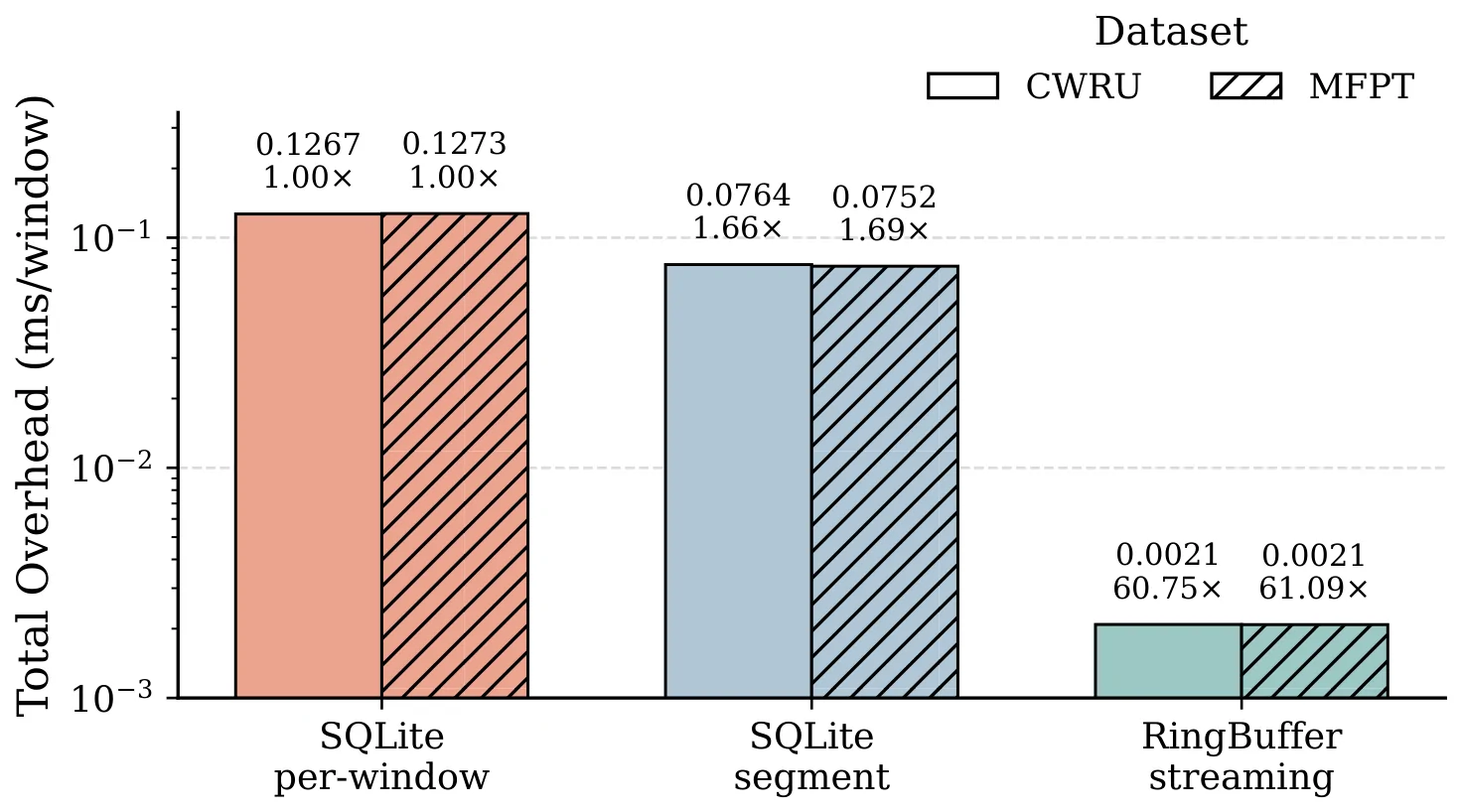
Front-end data access and window generation overhead of different input paths.

The overhead of different input paths was further evaluated to analyze the front-end cost before model inference. Table 4 reports the detailed results of data access time, window generation time, front-end overhead, and speedup for different input methods, while Figure 4 provides a visual comparison of the total overhead of the three input methods.

As shown in Table 4, SQLite per-window SELECT introduces the highest overhead among the three methods on both datasets. Since each sliding window is directly queried from the database, the total cost is determined by repeated database access. SQLite segment-level SELECT reduces this overhead by querying a continuous signal segment and then generating sliding windows in memory. Since the queried segment contains all samples required for the evaluated windows, the number of database queries is reduced from repeated per-window queries to a single segment query.

As shown in Table 4 and Figure 4, RingBuffer streaming achieves the lowest total overhead on both datasets. In this method, no additional database query is required during window extraction, and valid windows are directly generated from the buffer in memory. This makes RingBuffer more suitable for streaming sensor input, where high-frequency database queries may become a bottleneck before inference.

SQLite and RingBuffer have different input scenarios. SQLite is used for local historical data storage and query, while RingBuffer is designed for sensor streaming input. Therefore, this comparison is intended to quantify the overhead introduced by different input paths. The reported RingBuffer-based front-end speedup is calculated relative to the per-window SELECT baseline and reflects the reduction in repeated database-query overhead.

These results indicate that data access and window generation are important parts of the front-end overhead in the edge inference pipeline. Since these steps are executed on the CPU before NPU inference, reducing their overhead can lower the latency of input preparation. In this experiment, the RingBuffer-based path reduces the front-end overhead by avoiding repeated SQLite queries and generating windows directly from the in-memory buffer. Therefore, RingBuffer provides a lower-overhead streaming input path for the subsequent NPU inference stage.

### 5.3. Batch Inference Scalability

**Figure 5:**
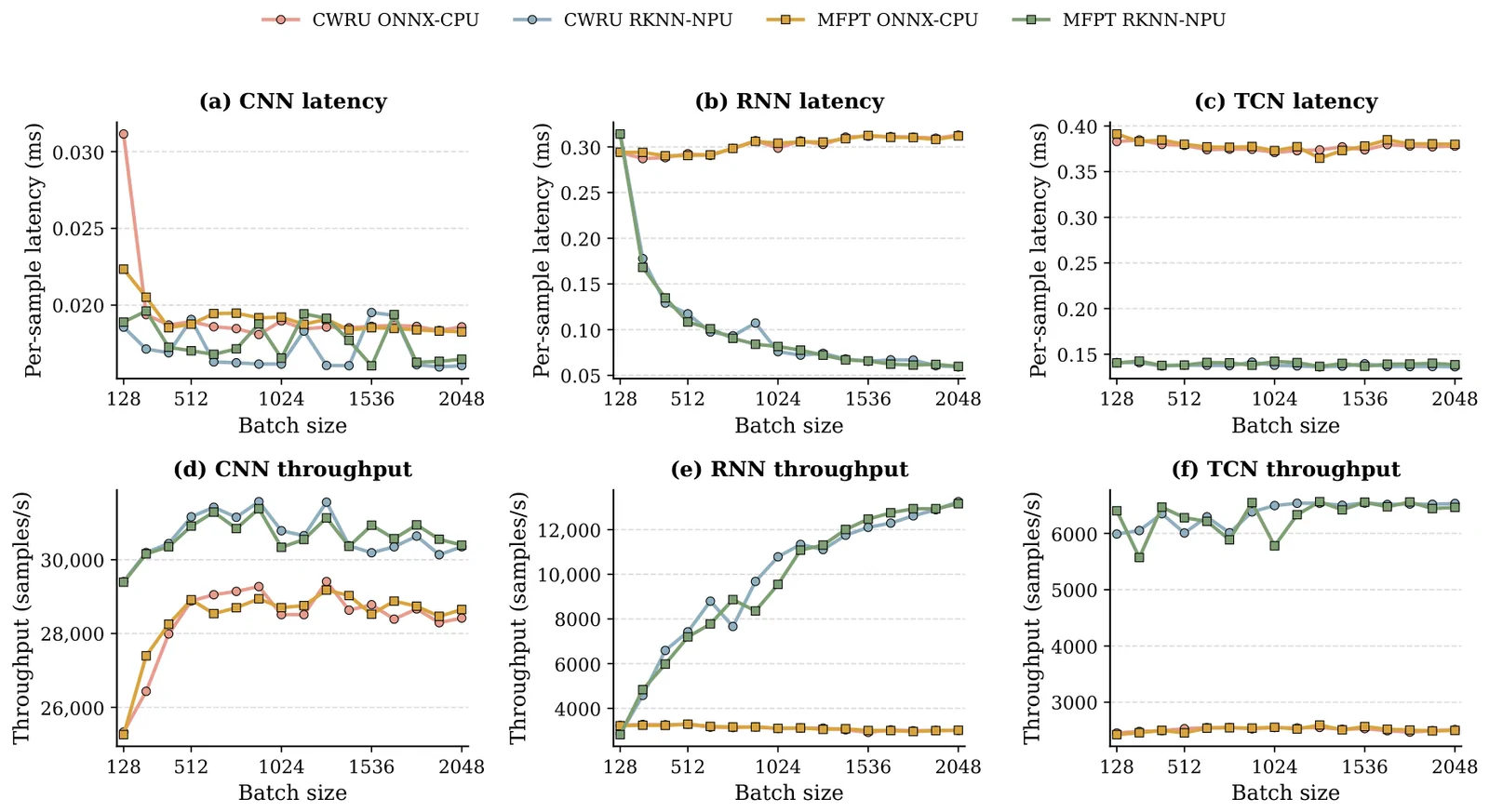
CPU–NPU inference scalability under different micro-batch sizes.

Figure 5 shows the inference scalability of CNN, RNN, and TCN models under different micro-batch sizes on the CWRU and MFPT datasets. The experiment compares ONNX-CPU and RKNN-NPU inference in terms of per-sample latency and throughput. The micro-batch size ranges from 128 to 2048. Some results in Figure 5 overlap because the corresponding results on the CWRU and MFPT datasets are very close. This indicates that the models exhibit similar batch inference behavior on the two datasets under the same runtime configuration.

For the CNN model, the per-sample latency of ONNX-CPU and RKNN-NPU is similar across most batch sizes on both datasets. RKNN-NPU shows lower latency and higher throughput in some cases, but the improvement does not increase clearly with the micro-batch size. This indicates that the CNN model is lightweight enough, and its inference cost is relatively low on both CPU and NPU, leaving limited room for further acceleration.

For the RNN model, the effect of NPU acceleration is more obvious. At small micro-batch sizes, RKNN-NPU does not have obvious improvement. But as the micro-batch size increases, the per-sample latency of RKNN-NPU decreases clearly, while the ONNX-CPU latency remains relatively stable. The throughput of RKNN-NPU also increases with the micro-batch size, showing better scalability than ONNX-CPU.

The TCN model shows a similar trend on both datasets. Compared with ONNX-CPU, RKNN-NPU achieves lower per-sample latency and higher throughput across the evaluated batch sizes. This indicates that the NPU can provide more effective acceleration for models with higher computational workload. The results from both RNN and TCN show that NPU acceleration is more significant when the model structure is more complex and the batch size is larger.

Overall, the results indicate that the effectiveness of NPU acceleration depends on both model structure and micro-batch configuration. The CNN model has limited acceleration potential in this experiment because of its low computational cost. In contrast, the RNN and TCN models benefit more from NPU execution when enough samples are grouped into a micro-batch. The trends observed on CWRU and MFPT are consistent, suggesting that the batch inference behavior is mainly affected by the model architecture, batch size and acceleration design.

The hardware utilization results further support this observation. In CPU-based inference, the CPU utilization is generally high, mostly around 80–100%, because the model computation is executed on the CPU. In contrast, RKNN-NPU inference significantly reduces CPU utilization. The CPU utilization is approximately 6–10% for CNN-NPU, 2–8% for RNN-NPU, and 1–6% for TCN-NPU. Meanwhile, the measured NPU utilization is about 15–17% for CNN-NPU, 24–27% for RNN-NPU, and 27–30% for TCN-NPU. These results indicate that the RKNN models are executed on the NPU during inference, and the higher NPU utilization of RNN and TCN is consistent with their stronger acceleration effect.

### 5.4. End-to-End Pipeline Performance

**Figure 6:**
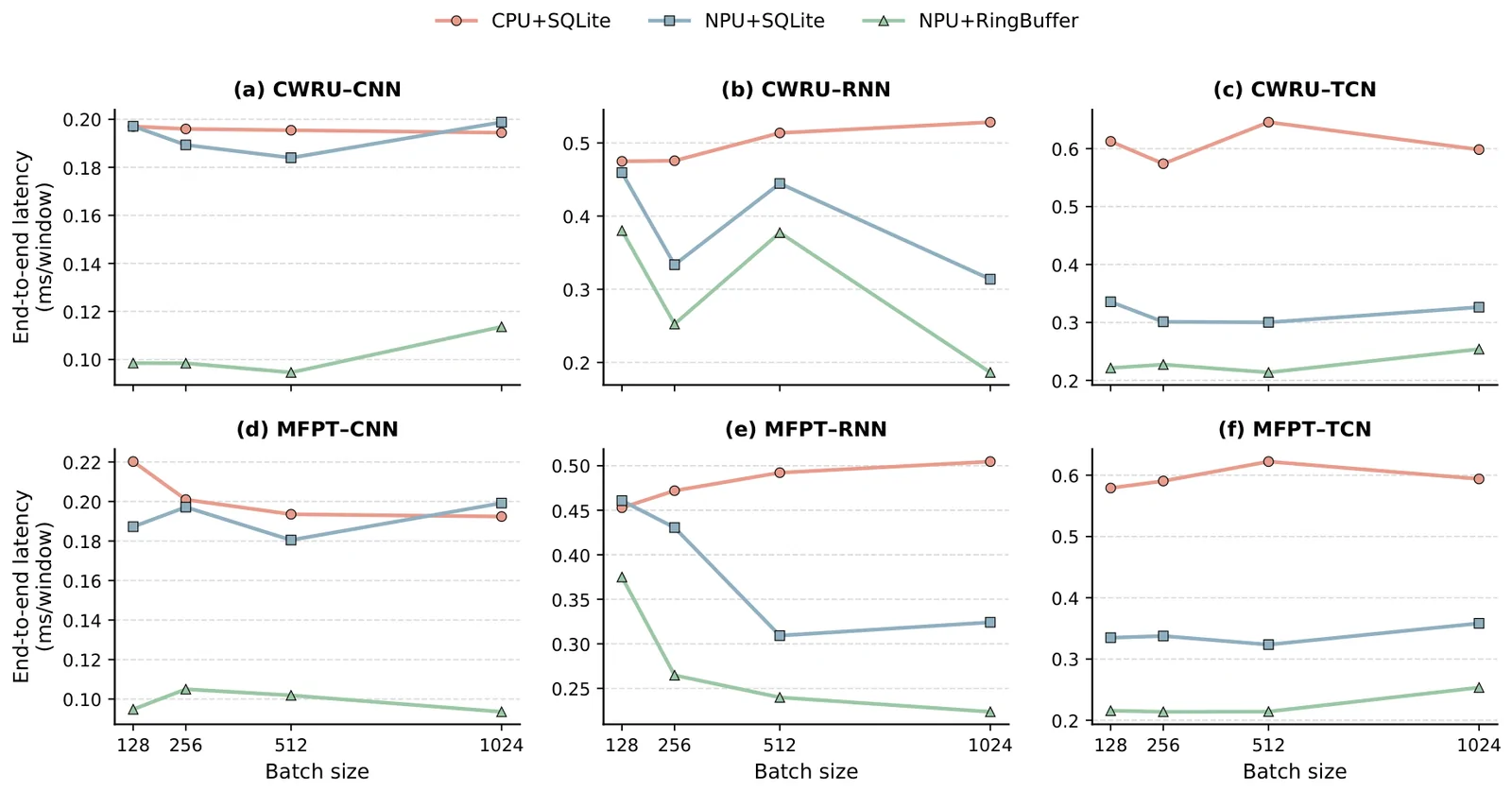
End-to-end latency of different micro-batch pipelines.

The end-to-end performance of the proposed deployment pipeline was evaluated under three configurations: CPU+SQLite, NPU+SQLite, and NPU+RingBuffer. Figure 6 shows the latency trends under different batch sizes on the CWRU and MFPT datasets, and Table 5 reports the corresponding latency and peak memory consumption. This experiment evaluates the complete processing path, including data access, window generation, micro-batch construction, input preparation, and model inference.

As shown in Figure 6, similar overall trends are observed on the two datasets. The NPU+RingBuffer pipeline generally achieves the lowest end-to-end latency across the three models. By replacing repeated SQLite queries with in-memory streaming window generation, the RingBuffer-based pipeline reduces the CPU-side preparation overhead before inference.

For the CNN model, the latency difference between CPU+SQLite and NPU+SQLite is relatively small, indicating that NPU execution alone does not bring a large latency reduction. However, NPU+RingBuffer reduces the CNN end-to-end latency by lowering the input-stage overhead, showing that the input path has a strong effect on the complete deployment pipeline.

For the RNN model, compared with CPU+SQLite, the NPU-based pipelines usually achieve lower latency, especially when the batch size increases. Among the three configurations, NPU+RingBuffer provides the lowest overall latency in most cases. This suggests that in this experiment, RNN inference benefits more from NPU execution and micro-batch processing than CNN inference.

For the TCN model, using NPU inference on both datasets reduces latency. Within the evaluated batch size range, the latency of NPU+RingBuffer is approximately 0.21–0.25 ms/window, while the latency of CPU+SQLite is approximately 0.57–0.65 ms/window.

Table 5 also reports the peak memory consumption of different pipeline configurations. For CNN, the NPU-based pipelines require more memory than CPU+SQLite, which may be related to the RKNN Runtime and NPU execution environment. For RNN, CPU memory consumption generally increases with the micro-batch size, while the NPU pipelines show stable peak memory. For TCN, the peak memory of both CPU and NPU pipelines increases with the micro-batch size. These results indicate that the memory behavior is model-dependent. NPU deployment introduces runtime memory overhead but can provide more stable memory usage for some model and batch configurations.

In conclusion, the end-to-end results show that the deployment performance is affected by both the inference backend and the input path. NPU execution is more useful for the RNN and TCN workloads, while RingBuffer streaming reduces CPU-side data preparation overhead for the three models. In this work, RingBuffer is used as a simple streaming buffer implementation, and its results show that reducing repeated data access and generating windows in memory can lower end-to-end latency in the CPU–NPU pipeline.

### 5.5. Cross-Platform Inference Performance and Energy Efficiency

Cross-platform inference experiments were conducted using three hardware configurations: an Intel Xeon CPU, an NVIDIA A100 GPU, and the RK3588 NPU. The CPU and GPU experiments were performed on the same server using ONNX Runtime, while the NPU experiments were conducted on the RK3588 device using RKNN Runtime. All experiments used a batch size of 128 and were executed continuously for 60 s. Table 6 reports the batch latency (Lat., ms), throughput (Thr., samples/s), CPU power ($P_{C}$, W), GPU power ($P_{G}$, W), total power ($P_{T}$, W), throughput per watt (Eff., samples/J), and energy consumption per sample ($E_{s}$, mJ/sample).

For the CPU-only configuration, $P_{T}$ is equal to the measured CPU power. For the GPU-accelerated configuration, $P_{T}$ is the sum of the measured CPU power and GPU power. For the RK3588 NPU, $P_{C}$ and $P_{G}$ are not applicable, and its power consumption was estimated as 8 W according to its maximum operating power. The throughput-per-watt and energy-per-sample values of the RK3588 platform were therefore calculated using this estimated power value.

The results show similar trends on both CWRU and MFPT datasets. The server CPU and GPU provide lower latency and higher throughput than the RK3588 NPU for the three models. However, both server configurations consume much more power than the RK3588 device. Their compute component power is also much higher than that of the RK3588 NPU. In addition, the lightweight fault-diagnosis models cannot make full use of the A100 GPU during inference, so the GPU configuration exhibits lower component power than the CPU-only configuration in some cases.

Overall, the server devices achieve the lowest latency and the highest throughput for the evaluated models. Although the GPU provides lower latency and higher throughput, deploying such a platform requires higher cost and power consumption, which is not suitable for resource-constrained edge scenarios. In contrast, the RK3588 achieves lower cost and has competitive energy efficiency. Therefore, the RK3588 platform provides a practical choice for industrial edge fault diagnosis scenarios with low deployment cost, power consumption, and local inference capability.

## 6. Discussion

The model consistency results show that the trained CNN, RNN, and TCN models maintain acceptable diagnostic performance after ONNX-to-RKNN conversion on the RK3588 NPU across the CWRU and MFPT datasets.

The batch inference scalability results further indicate that NPU acceleration is not equally effective for all models. Compared with the CNN workload, the RNN and TCN workloads generally benefit more from NPU execution at suitable micro-batch sizes. Therefore, model structure and micro-batch configuration should be considered during edge deployment.

For online deployment, a larger micro-batch may introduce additional waiting time, because a window has to remain in the batch buffer until enough windows are accumulated. Therefore, batch size selection should consider not only throughput, but also the sampling rate and response-time requirement of the target application.

CPU-side data preparation also affects the end-to-end latency of the CPU–NPU heterogeneous framework. The results suggest that input-side buffering and in-memory window generation can reduce the preparation overhead before NPU inference.

This work still has some limitations. The experiments were conducted on one RK3588-based edge platform and two bearing fault datasets for the three models. Therefore, the results mainly demonstrate the feasibility and runtime behavior of the proposed framework under the tested deployment settings. In addition, the RingBuffer used in this work is a simple fixed-size streaming buffer. It is effective for reducing repeated database access in the tested pipeline, but it is not the only possible input management strategy. Future work will evaluate the framework on more industrial datasets, different edge platforms, more complex fault diagnosis models, and more advanced input buffering strategies.

## 7. Conclusions

This paper developed a CPU–NPU heterogeneous edge fault diagnosis framework for industrial sensor data. In the proposed framework, the CPU performs local data access, buffering, sliding window generation, and micro-batch preparation, while the NPU executes the converted RKNN fault diagnosis models through the RKNN Runtime. CNN, RNN, and TCN models were deployed on an RK3588-based edge device and evaluated on the CWRU and MFPT datasets in terms of model conversion consistency, data access overhead, micro-batch inference scalability, and end-to-end pipeline performance.

The experimental results show that the converted RKNN models can maintain acceptable diagnostic performance compared with ONNX-based CPU inference. The RingBuffer-based streaming input shows that input-side buffering and in-memory window generation can reduce CPU-side data preparation overhead, while NPU acceleration is more effective for the RNN and TCN models under larger micro-batch sizes. Similar overall latency trends are observed on the CWRU and MFPT datasets, indicating that the benefits of the framework are not limited to a single dataset or model architecture. These results indicate that both the input path and the micro-batch configuration affect latency, throughput, and CPU-side workload in edge deployment. Overall, the proposed framework provides a useful reference for local fault diagnosis on resource-constrained CPU–NPU edge devices.

## Figures and Tables

**Table 1:** Key structural configurations of the evaluated models.

Model	Main Structure	Channels	Kernel Size	Hidden Units	Dropout (CWRU/MFPT)
CNN	2 Conv1D + 2 FC	32, 64	3	128	–
RNN	1 LSTM + 1 FC	–	–	128	0.3/0.3
TCN	3 TCN residual blocks + 1 FC	32, 64, 128	3	–	0.2/0.0

**Table 2:** Training configurations and model file sizes for different datasets and models.

Dataset	Model	Learning Rate	Epochs	ONNX Size	RKNN Size
CWRU	CNN	0.001	25	1.03 MB	1.66 MB
	RNN	0.001	100	0.26 MB	1.11 MB
	TCN	0.0001	50	0.42 MB	10.27 MB
MFPT	CNN	0.001	200	1.03 MB	1.70 MB
	RNN	0.001	1000	0.26 MB	1.10 MB
	TCN	0.001	200	0.41 MB	10.30 MB

**Table 3:** Model conversion consistency between ONNX-CPU and RKNN-NPU inference on the CWRU and MFPT datasets.

Dataset	Model	ONNX Acc.	RKNN Acc.	ONNX F1	RKNN F1	Consistency	Inconsistent Pred.
CWRU	CNN	93.84%	93.84%	93.27%	93.27%	100.00%	0
	RNN	97.37%	97.12%	97.12%	96.84%	99.29%	14
	TCN	98.94%	98.94%	98.84%	98.84%	100.00%	0
MFPT	CNN	94.00%	93.95%	94.00%	93.95%	99.95%	1
	RNN	96.38%	96.38%	96.38%	96.38%	99.43%	12
	TCN	96.86%	96.86%	96.86%	96.86%	100.00%	0

**Table 4:** Data access and window generation overhead of different input paths.

Dataset	Method	Data Access (ms/Window)	Window Gen. (ms/Window)	Front-End Overhead (ms/Window)	Front-End Speedup (×)
CWRU	SQLite per-window	0.1267	–	0.1267	1.00
	SQLite segment	0.0760	0.0004	0.0764	1.66
	RingBuffer streaming	–	0.0021	0.0021	60.75
MFPT	SQLite per-window	0.1273	–	0.1273	1.00
	SQLite segment	0.0748	0.0004	0.0752	1.69
	RingBuffer streaming	–	0.0021	0.0021	61.09

**Table 5:** End-to-end latency and memory of micro-batch pipelines.

Dataset	Model	Batch	CPU+SQLite	NPU+SQLite	NPU+RingBuffer
			Latency (ms/Window)/Peak Memory (MB)		
CWRU	CNN	128	0.1970/246.7	0.1971/592.7	0.0985/590.9
		256	0.1960/255.2	0.1893/593.0	0.0984/590.9
		512	0.1955/268.3	0.1840/593.2	0.0946/591.4
		1024	0.1944/268.8	0.1988/593.0	0.1136/590.9
	RNN	128	0.4749/315.0	0.4594/500.5	0.3802/255.1
		256	0.4757/358.5	0.3336/500.5	0.2525/255.1
		512	0.5137/540.0	0.4446/499.7	0.3775/500.5
		1024	0.5284/639.6	0.3139/500.3	0.1864/499.7
	TCN	128	0.6125/292.9	0.3356/272.4	0.2215/272.1
		256	0.5740/369.0	0.3011/307.5	0.2274/305.4
		512	0.6457/498.1	0.3002/376.4	0.2138/374.6
		1024	0.5984/596.7	0.3264/515.2	0.2540/513.3
MFPT	CNN	128	0.2202/252.5	0.1873/612.4	0.0948/610.0
		256	0.2010/262.3	0.1972/612.4	0.1050/610.3
		512	0.1936/274.4	0.1805/612.8	0.1019/610.8
		1024	0.1924/274.8	0.1992/612.6	0.0936/610.6
	RNN	128	0.4528/326.3	0.4608/513.0	0.3750/513.0
		256	0.4720/368.2	0.4305/512.4	0.2648/512.4
		512	0.4922/551.3	0.3093/512.4	0.2399/512.4
		1024	0.5046/914.4	0.3242/512.4	0.2237/512.4
	TCN	128	0.5791/314.9	0.3348/290.2	0.2157/290.6
		256	0.5906/383.8	0.3376/323.6	0.2139/323.6
		512	0.6224/513.3	0.3236/393.8	0.2143/391.7
		1024	0.5940/611.8	0.3583/532.0	0.2536/530.4

**Table 6:** Cross-platform comparison of inference performance and energy efficiency.

Dataset	Model	Platform	Lat.	Thr.	$P_{C}$	$P_{G}$	$P_{T}$	Eff. ↑	$E_{s}↓$
CWRU	CNN	Xeon CPU	0.362	137,490.8	315.9	–	315.9	435.2	2.298
		A100 GPU	0.217	168,305.7	176.6	53.1	229.7	732.7	1.365
		RK3588	2.599	27,863.2	–	–	8	3482.9	0.287
	RNN	Xeon CPU	1.519	61,063.0	394.8	–	394.8	154.7	6.465
		A100 GPU	1.576	60,027.9	179.3	93.0	272.3	220.5	4.536
		RK3588	39.934	2844.4	–	–	8	355.6	2.812
	TCN	Xeon CPU	6.276	18,642.3	348.6	–	348.6	53.5	18.701
		A100 GPU	0.546	116,218.8	177.4	116.9	294.4	394.8	2.533
		RK3588	18.029	5989.4	–	–	8	748.7	1.336
MFPT	CNN	Xeon CPU	0.351	140,107.4	316.2	–	316.2	443.1	2.257
		A100 GPU	0.205	172,157.7	175.9	53.6	229.4	750.5	1.333
		RK3588	2.419	29,392.7	–	–	8	3674.1	0.272
	RNN	Xeon CPU	1.460	62,891.7	397.6	–	397.6	158.2	6.322
		A100 GPU	1.570	60,400.5	176.3	94.4	270.7	223.1	4.482
		RK3588	40.204	2821.8	–	–	8	352.7	2.835
	TCN	Xeon CPU	6.305	18,556.5	347.5	–	347.5	53.4	18.727
		A100 GPU	0.536	117,604.6	175.8	121.9	297.7	395.1	2.531
		RK3588	17.991	6402.2	–	–	8	800.3	1.250

Note: ↑ indicates that a higher value is better, and ↓ indicates that a lower value is better.

## Data Availability

Publicly available datasets were analyzed in this study. The CWRU Bearing Data Center dataset is available at https://engineering.case.edu/bearingdatacenter/download-data-file, and the MFPT bearing dataset is available at https://www.mfpt.org/fault-data-sets/.

## References

[1] Lei Y., Li N., Guo L., Li N., Yan T., Lin J. (2018). Machinery health prognostics: A systematic review from data acquisition to RUL prediction. Mech. Syst. Signal Process..

[2] Laghari R.A., Memon S., Ansari M., Shah W., Shakir M., Khan A.A., Hussain B., Laghari A.A. (2026). Artificial Intelligence Based Contemporary Computational Paradigms for Intelligent Machining Processes: Review on Assessment, Progress and Recent Development. Arch. Comput. Methods Eng..

[3] Neupane D., Seok J. (2020). Bearing fault detection and diagnosis using case western reserve university dataset with deep learning approaches: A review. IEEE Access.

[4] Cortes C., Vapnik V. (1995). Support-vector networks. Mach. Learn..

[5] MacQueen J. (1967). Some methods for classification and analysis of multivariate observations. Proceedings of the Fifth Berkeley Symposium on Mathematical Statistics and Probability, Volume 1: Statistics.

[6] LeCun Y., Bottou L., Bengio Y., Haffner P. (1998). Gradient-based learning applied to document recognition. Proc. IEEE.

[7] Rumelhart D.E., Hinton G.E., Williams R.J. (1986). Learning representations by back-propagating errors. Nature.

[8] Vaswani A., Shazeer N., Parmar N., Uszkoreit J., Jones L., Gomez A.N., Kaiser Ł., Polosukhin I. (2017). Attention is all you need. Advances in Neural Information Processing Systems 30.

[9] Xu K., Li C., Dong W., Xing C., Sun Y. Comparison of Traditional and Large Model Methods for Bearing Fault Classification Tasks. Proceedings of the 2025 Global Reliability and Prognostics and Health Management Conference (PHM-Xian).

[10] Lu S., Lu J., An K., Wang X., He Q. (2023). Edge computing on IoT for machine signal processing and fault diagnosis: A review. IEEE Internet Things J..

[11] Wang J., Xu C., Zhang J., Zhong R. (2022). Big data analytics for intelligent manufacturing systems: A review. J. Manuf. Syst..

[12] Jing T., Tian X., Hu H., Ma L. (2021). Deep learning-based cloud–edge collaboration framework for remaining useful life prediction of machinery. IEEE Trans. Ind. Inform..

[13] Qu G., Chen Q., Wei W., Lin Z., Chen X., Huang K. (2025). Mobile edge intelligence for large language models: A contemporary survey. IEEE Commun. Surv. Tutor..

[14] Bala A., Rashid R.Z.J.A., Ismail I., Oliva D., Muhammad N., Sait S.M., Al-Utaibi K.A., Amosa T.I., Memon K.A. (2024). Artificial intelligence and edge computing for machine maintenance-review. Artif. Intell. Rev..

[15] Nayana B.R., Geethanjali P. (2017). Analysis of Statistical Time-Domain Features Effectiveness in Identification of Bearing Faults From Vibration Signal. IEEE Sens. J..

[16] Juodelyte D., Cheplygina V., Graversen T., Bonnet P. Predicting bearings degradation stages for predictive maintenance in the pharmaceutical industry. Proceedings of the 28th ACM Sigkdd Conference on Knowledge Discovery and Data Mining.

[17] Tian J., Morillo C., Azarian M.H., Pecht M. (2015). Motor bearing fault detection using spectral kurtosis-based feature extraction coupled with K-nearest neighbor distance analysis. IEEE Trans. Ind. Electron..

[18] Senanayaka J.S.L., Van Khang H., Robbersmyr K.G. (2020). Toward self-supervised feature learning for online diagnosis of multiple faults in electric powertrains. IEEE Trans. Ind. Inform..

[19] Pan H., He X., Tang S., Meng F. (2018). An improved bearing fault diagnosis method using one-dimensional CNN and LSTM. Stroj. Vestn.-J. Mech. Eng..

[20] Jin Y., Hou L., Chen Y. (2022). A time series transformer based method for the rotating machinery fault diagnosis. Neurocomputing.

[21] Liu Y., Wang Y., Yang K., Sun P., Liu J., Du Y., Wu J., Boukerche A., Leung V.C., Hu X. (2025). Edge-Cloud Collaborative Computing on Distributed Intelligence and Model Optimization: A Survey. arXiv.

[22] Pham M.T., Kim J.M., Kim C.H. (2020). Deep Learning-Based Bearing Fault Diagnosis Method for Embedded Systems. Sensors.

[23] Lu S., Qian G., He Q., Liu F., Liu Y., Wang Q. (2020). In Situ Motor Fault Diagnosis Using Enhanced Convolutional Neural Network in an Embedded System. IEEE Sens. J..

[24] Huang Y., Liang S., Cui T., Mu X., Luo T., Wang S., Wu G. (2024). Edge Computing and Fault Diagnosis of Rotating Machinery Based on MobileNet in Wireless Sensor Networks for Mechanical Vibration. Sensors.

[25] Gong R., Wang C., Li J., Xu Y. (2023). Lightweight fault diagnosis method in embedded system based on knowledge distillation. J. Mech. Sci. Technol..

[26] Nguyen H.A.H., Kim C.H. (2025). Efficient Bearing Fault Diagnosis for Edge Computing Using Grayscale Spectrograms and Hybrid Neural Model Compression. IEEE Access.

[27] Zhou Z., Qiao Y., Lin X., Li P., Wu N., Yu D. (2025). A Deployment Method for Motor Fault Diagnosis Application Based on Edge Intelligence. Sensors.

[28] Jayanth R., Gupta N., Prasanna V. (2024). Benchmarking Edge AI Platforms for High-Performance ML Inference. arXiv.

[29] Qian M., Wang Y., Liu S., Xu Z., Ji Z., Chen M., Wu H., Zhang Z. (2025). Real time wire rope detection method based on Rockchip RK3588. Sci. Rep..

[30] ONNX Runtime Developers (2021). ONNX Runtime. Version: 1.23.2. https://onnxruntime.ai/.

[31] RKNN Toolkit2. Version: 2.3.2. https://github.com/airockchip/rknn-toolkit2.

[32] Case Western Reserve University Bearing Data Center (2012). CWRU Dataset. https://engineering.case.edu/bearingdatacenter/download-data-file.

[33] Machinery Failure Prevention Technology Society. https://www.mfpt.org/fault-data-sets/.

[34] Bai S., Kolter J.Z., Koltun V. (2018). An Empirical Evaluation of Generic Convolutional and Recurrent Networks for Sequence Modeling. arXiv.

